# A study of the incidence of BCG vaccine complications in infants of Babol, Mazandaran (2011-2013)

**Published:** 2016

**Authors:** Rahim Barari-Savadkouhi, Azin Shour, Jila Masrour-Roudsari

**Affiliations:** 1 Tropical Medicine and Infection Diseases Research Center, Babol University of Medical Sciences, Babol, Iran.; 2 Deputy of Health, Babol University of Medical Sciences, Babol Iran.

**Keywords:** BCG vaccination, Complications, Lymphadenitis, Disseminated TB, Urine discoloration

## Abstract

**Background:**

BCG vaccination which is administered to prevent tuberculosis is sometimes associated with serious complications. This study aimed to determine the incidence of complications of BCG vaccination in Babol.

**Methods:**

All infants who received BCG vaccination between 2011-2013 in health centers of Babol entered the study. Data regarding complications of vaccine were extracted according to the National Inventory of babies. All complicated cases were confirmed by the Academic Committee to review the adverse consequences of the vaccine.

**Results:**

Among the 15984 vaccinated neonates, 150 (0.93%) cases presented lymphadenitis. 46.5% were females and 53.5% were males; 43% were rural residents and 57% were urban residents. No cases of lymphadenitis including 1% of lymphadenitis with abscess formation were recovered without treatment. Disseminated infection occurred in 3 cases of immune deficient patients who responded to the treatment. Most complications occurred during 4 months after vaccination.

**Conclusion:**

According to the results of this study, the prevalence of lymphadenitis in Babol was higher than the standard of WHO. This may be attributed to type and vaccine storage and injection technique. These findings justify further training of health-center workers.

BCG vaccination is recommended to prevent the ourrence of tuberculosis across the world, particularly for prevention of Bn and disseminated TB. In Iran, BCG vaccination is performed in the neonatal age, especially at birth, the bacteria used for vaccine is Mycobacterium bovis with a concentration of 1 mg ml (1). Administration of BCG vaccine decreases the risk factors of pulmonary and extrapulmonary tuberculosis by 50%. Adequate response following BCG vaccination is defined as the appearnce of a red zone at the injection site, followed by the development of a wound and a scab is formed after about 6 weeks with consequent permanent small scar. Enlargement of lymph node is normal effect of vaccination (2). Adverse reactions to BCG vaccination at the injection site is well-known (3). The adverse effects are divided in two categories of moderate and severe lesions. The moderate lesions are local and the most common is lymphadenitis. Skin complications such as eczema and lopoid reaction also belonged to the medium category. The incidence of these complications is less than one in a thousand (4). 

Severe complications include suppurative lymphadenitis, osteomyelitis, osteitis and disseminated infection caused by the vaccine and the global incidence has been 100-1000 and 1-700, and 2 in a million, respectively (5). Most complications from vaccine occur when there are changes in the species of bacteria used in the vaccine or by its low percentage as a result of laboratory errors or vaccine storage and injection technique, the lack of vaccine’s cold chain and inappropriate vaccine doses (6). The complications are more common in people who have a history of previous exposure to tuberculosis cases (7), and in particular, the incidence of complications.

Recent observations have indicated higher than expected number of patients who present to health center clinics because of complications of BCG vaccination. For these reasons, the present study was conducted to investigate the frequency of major complications caused by BCG vaccine in children under the full coverage of health centers of Babol, Mazandaran.

## Methods

The population of this study included all children born in 2012-2013 who have received BCG vaccination in Babol. After vaccine-injection, the parents of the vaccinated children were asked to inform regarding the development of any local or systemic manifestations such as swelling, nausea, vomiting, any change in the color of urine, restlessness and frequent urination immediately to health centers. All vaccinated children were followed-up after vaccination and examined by a physician or experts in health centers two weeks after vaccination, then monthly for 3 months until recovery of side effects. Data were obtained from the checklist extracted from the National Inventory in Form 2 and were sent to Communicable DisEASE Prevention and Control Unit. The sent items were discussed in vaccine committee of its adverse effects in the presence of experts among the faculty members; after the confirmation of the effects of the BCG vaccine, questionnaires were approved in the study plan.

## Results

In this study, 150 out of 15,984 vaccinated neonates (0.93%) presented with Lymphadenitis among which 70 (46.7 %) cases were females and 80 (35.3%) males; 65 cases (43%) were rural residents and 85 (57%) were urban, residents. 99% with lymphadenopathy, lymphadenitis, 1% abscess, in which 99.8 cases recovered without intervention (drug, drainage or surgery) and 3 (0.02 %) cases, including two girls and one boy received diet therapy for 12 months because they had disseminated infection due to a severe immune deficiency; diet therapy included: two months with isoniazid, rifampin, ethambutol, clarithromycin and ten months with isoniazid and rifampin. 

In 159 (0.1 %) cases the appearance of urine changed to orange color on the second day after vaccination and recovered after a week. 31 (0.2%) cases had severe restlessness 48 hours to 10 days after injection. 48 (0.3%) cases had nausea and vomiting from the second day of birth to 2 weeks and in the followed examinations, some had cases of GERD who were referred for treatment while the others recovered.

In terms of interval between onsets of symptoms, most complications developed 2-4 after vaccination. The onset of complications at different ages are shown in table 1.

**Table 1:** Prevalence of complications of BCG vaccine at different ages

**Age (Months) **	**≥** **2**	**2-4**	**4-6**	**6-8**	**≤** **8**
Complication level (%)	24	35	26	9	6

Prevalence of complications varied from 0.6 % to 1.7% across different health cares 

## Discussion

The results of this study indicate a low prevalence of complications after BCG vaccination with rare severe adverse reactions (8). In this study, disseminated TB infection occurred in children with history of immune deficiency (9 and 10) although there are also reports of disseminated infection in healthy individuals (11). Others reported adverse reactions that included lymphadenitis, erythema, stiffness and abscess at the injection site and lymphadenopathy (12 and 13). In this study, the most common complication was lymphadenitis which was observed similar to other studies, but greater than the standard of WHO (2, 3, 5, 14, 15). Lymphadenitis in the under an area which does not need treatment and can recover by itself. Our cases with immune deficiency were similar to other studies treated and recovered by drugs like other studies (1, 16).

The adverse effects as a result of the BCG vaccine were also dependent on the type of vaccine. In our study, vaccine type was **(), **which like other studies, it was observed that lymphadenitis occurs in the SSI BCG vaccine more than the other types (17, 12). Previous studies, showed the occurrence of some complications. Within 2 years after vaccination. Nonetheless, most of these complications occurred in the first 20 weeks after vaccine-injection (12); these results were similar to the results of a recent study in which the most effects have been seen within 2-4 months which can be attributed to the lack of T helper 1 in the early neonatal period.

Other complications reported in the current study was the development of orange color urine which was not reported in other studies. The cause of urine discoloration in the present study was detected and remained to clear. However, it was not a serious complication and was self-limiting. In this study, the incidence of complications varied across different health centers, which can be attributed to inappropriate dosages, storage and types of vaccine and variations in vaccination injection techniques (2). 

In conclusion, the results of this study indicate that the development of complications particularly lymphadenitis in health care centers of Babol is higher than expected and suggests further training of health center staff regarding injection techniques, storage type of vaccine and replacing it with a vaccine with fewer side effects.

## Notes

**Funding:** Self-funded.
